# Hypoxia-targeted BOLD MRI signal heterogeneity as a complementary metric for glioblastoma characterization: a pilot study

**DOI:** 10.1007/s11060-026-05762-6

**Published:** 2026-08-20

**Authors:** Leonie Zerweck, Tristan Schmidlechner, Vittorio Stumpo, Elisabeth Jehli, Meltem Gönel, Flavia Müller, Jacopo Bellomo, Martina Sebök, Andrea Bink, Zsolt Kulcsár, Michael Weller, Luca Regli, Christiaan H.B. van Niftrik, Jorn Fierstra

**Affiliations:** 1 Department of Neurosurgery, Clinical Neuroscience Center, University Hospital Zurich and University of Zurich, Zurich, Switzerland; 2 Department of Diagnostic and Interventional Neuroradiology, University Hospital Tuebingen, Tuebingen, Germany; 3 Department of Neuroradiology, Clinical Neuroscience Center, University Hospital Zurich and University of Zurich, Zurich, Switzerland; 4 Department of Neurology, Clinical Neuroscience Center, University Hospital Zurich and University of Zurich, Zurich, Switzerland

**Keywords:** Brain tumor, Oxygenation, Angiogenesis, Biomarker, Microenvironment

## Abstract

**Purpose:**

Hypoxia-targeted blood oxygen level–dependent (BOLD) MRI is an emerging non-contrast imaging technique for characterizing tumor biology in isocitrate dehydrogenase-wildtype glioblastoma. Beyond the magnitude of BOLD signal changes, signal heterogeneity may provide valuable information about tumor aggressiveness and microenvironmental complexity. The aim was to assess both the magnitude and heterogeneity of hypoxia-targeted BOLD signal changes across subregions of glioblastomas.

**Methods:**

In this prospective pilot study, hypoxia-targeted BOLD MRI data from 12 patients with histopathologically confirmed glioblastoma were analyzed. Tumor subregions were defined using an automated segmentation framework (Oncohabitats) based on conventional MRI and dynamic susceptibility contrast (DSC) MR perfusion imaging. Voxel-wise BOLD signal changes were calculated within the following volumes of interest: highly angiogenic tumor (HAT), low angiogenic tumor (LAT), peritumoral edema, and mirrored contralateral control tissue (mCET). The magnitude of BOLD signal changes was assessed using median BOLD signal changes, while heterogeneity was quantified using interquartile ranges (IQR), and kernel density estimation-derived entropy. Friedman tests with Dunn post-hoc tests were used to assess parameter differences between the VOIs.

**Results:**

HAT demonstrated the largest BOLD signal changes compared with LAT, edema, and mCET (all FDR-adjusted *p* ≤ 0.017). Heterogeneity metrics revealed significantly increased IQR and entropy in HAT compared with edema and mCET (all FDR-adjusted *p* < 0.008), with LAT showing intermediate values.

**Conclusion:**

Hypoxia-targeted BOLD MRI reveals both greater BOLD signal changes and elevated intratumoral heterogeneity in highly angiogenic and likely aggressive glioblastoma subregions. These findings suggest that heterogeneity metrics may provide complementary information to BOLD signal magnitude alone.

**Supplementary Information:**

The online version contains supplementary material available at https://doi.org/10.1007/s11060-026-05762-6.

## Introduction

Hypoxia-targeted blood oxygen level–dependent (BOLD) MRI has recently emerged as a promising contrast agent-free imaging technique in neuro-oncology [1–3]. It may allow for the delineation of biologically aggressive tumor subregions and the detection of infiltrative tumor spread beyond contrast-enhancing margins, which could be relevant for guiding supramarginal resection strategies and optimizing radiotherapy planning [4].

Controlled inhalation of a hypoxic, isocapnic gas mixture increases deoxyhemoglobin levels, which act as an endogenous contrast agent due to the different magnetic susceptibility of deoxyhemoglobin and oxyhemoglobin, resulting in a global BOLD signal decrease [5]. Owing to the maintenance of isocapnia and the relatively weak vasoactive properties of oxygen [6, 7], relevant vasodilatory effects are not expected. Consequently, regional variations in the BOLD signal may reflect differences in vascular density, baseline perfusion, and the unmasking of hypoxic and acidic microenvironments. Although tumor tissue is often hypoxic and acidic [8, 9], BOLD reflects deoxyhemoglobin-sensitive susceptibility effects rather than baseline tissue pO_2_ alone. In brain tumors, BOLD is additionally influenced by blood flow, blood volume, hemoglobin saturation, and oxygen dissociation dynamics, so the relationship to hypoxia seems to be multifactorial. Tumor-associated acidosis [9] might, via the Bohr effect, shift the hemoglobin-oxygen dissociation curve rightward, reducing hemoglobin’s oxygen affinity and favoring oxygen unloading [10, 11]. This mechanism could increase the amount of additional deoxyhemoglobin formed for a given further reduction in oxygenation, rather than reflecting an already-saturated baseline. The hypoxia-induced BOLD signal decrease might therefore be amplified in tumor tissue relative to physiological tissue.

Isocitrate dehydrogenase-wildtype glioblastoma is the most common and aggressive primary brain tumor [12, 13]. It is characterized by diffuse infiltration, extensive neoangiogenesis, and a profoundly hypoxic tumor microenvironment [12–15]. In line with these pathophysiological features, glioblastoma tissue exhibits a more pronounced hypoxic-induced BOLD signal decrease than healthy tissue [2]. Another hallmark of isocitrate dehydrogenase-wildtype glioblastoma is its significant intratumoral heterogeneity [16–18], which is evident at both the molecular and macroscopic levels [18]. This heterogeneity extends to the tumor vasculature itself, which is architecturally and functionally abnormal, with convoluted vasculature, arteriovenous shunting, and dysfunctional neovascularization, resulting in spatially variable vascular density, perfusion, and oxygenation status within a given tumor [3, 19, 20]. Because more aggressive tumor regions have been reported to show more disorganized vascular remodeling and heterogeneous angiogenesis, and because hypoxia-targeted BOLD signal is sensitive to regional vascular density and oxygenation [19, 21, 22], we hypothesized that this same underlying biological heterogeneity might be reflected as spatial variability in the BOLD signal response, potentially more pronounced in more aggressive tumor subregions.

While previous studies have primarily focused on the magnitude of the hypoxia-targeted BOLD signal change, it remains unclear whether tumor aggressiveness is also reflected in its spatial BOLD signal heterogeneity. Here, we conceptualize signal heterogeneity as the degree of voxel-wise variability in hypoxia-targeted BOLD signal change within a tumor subregion, reflecting the presence of physiologically or microstructurally distinct sub-compartments – such as areas of differing vascular density, oxygen extraction, or admixed necrotic and viable tumor tissue – rather than a single, homogeneous tissue response [16–18]. Therefore, the aim of this pilot study was to assess both the magnitude and heterogeneity of the hypoxia-targeted BOLD signal changes across tumor subregions with varying degrees of vascularization, as defined by an automated segmentation framework based on morphological MRI and dynamic susceptibility contrast (DSC) MR perfusion imaging.

## Methods

### Study design

This prospective single center study was performed in accordance with the principles of the Declaration of Helsinki and approved by the ethics board of the Canton of Zurich, Switzerland (ID number 2020–02314). Written informed consent was obtained from all participants prior to inclusion in the study.

### Patients

Patients with suspected glioblastoma who were scheduled for biopsy or resection were prospectively enrolled between 2024 and 2026 and underwent BOLD MRI with transient hypoxic stimulus. Final inclusion criteria comprised histopathologically confirmed diagnosis of isocitrate dehydrogenase-wildtype glioblastoma and the availability of DSC MR perfusion acquired as part of routine clinical imaging within a maximum interval of two weeks. Exclusion criteria included prior neurosurgical therapy, known severe cardiopulmonary disease, contraindications to MRI (e.g., pacemaker, metallic implants, or severe claustrophobia), age < 18 years, and inability or unwillingness to provide informed consent.

As this study was designed as a pilot study, the target sample size reflected the feasible yield within a realistic recruitment period at a single center, rather than a prospectively pre-specified target based on a formal a priori power calculation. In total, 16 patients were enrolled. Three patients were excluded after histopathological review because the diagnosis of isocitrate dehydrogenase-wildtype glioblastoma was not confirmed. One data set was excluded from the further analysis due to insufficient segmentation. Patient characteristics are presented in Table 1.

**Table 1:** Patient characteristics

Patient Number	Age	Sex	Diagnosis including moleculargenetic results	Treatment at BOLD MRI examination^a^	Karnofsky Performance Status [%]
1	57	male	GBM, IDH-wildtype, ATRX-wildtype, TP53-wildtype, MGMT unmethylated, EGFR amplification, CDKN2A/B deletion, + 7/−10	Levetiracetam	n.a.; ECOG score 1^1^
2	64	male	GBM, IDH-wildtype, ATRX-wildtype, TP53 mutation, MGMT methylated, GFRA amplification, CDK4 amplification, PTEN deletion, + 7/−10	Dexamethasone	80%
3	56	male	GBM, IDH-wildtype, ATRX-wildtype, MGMT unmethylated, EGFR amplification, + 7/−10	Levetiracetam, lacosamide, perampanel	90%
4	67	female	GBM, IDH-wildtype, ATRX-wildtype, MGMT methylated, EGFR amplification, CDKN2A/B deletion, + 7/−10	Dexamethasone	80%
5	47	male	GBM, IDH-wildtype, ATRX-wildtype, TP53 mutation, MGMT unmethylated	-	90%
6	63	male	GBM, IDH-wildtype, ATRX-wildtype, TP53 mutation, MGMT methylated, RB1 deletion, + 7/−10	Dexamethasone, levetiracetam	90%
7	65	male	GBM, IDH-wildtype, ATRX-wildtype, MGMT not assessable, CDKN2A/B deletion, + 7/−10	Dexamethasone, clonazepam	90%
8	59	female	GBM, IDH-wildtype, ATRX-wildtype, TP53 mutation, MGMT unmethylated, + 7/−10	Dexamethasone, levetiracetam	70%
9	67	male	GBM, IDH-wildtype, ATRX-wildtype, H3k27me3 retained, MGMT methylated, EGFR amplification, MDM2 amplification, CDKN2A/B deletion, PTEN deletion	Dexamethasone, levetiracetam	80%
10	49	male	GBM, IDH-wildtype, ATRX-wildtype, TP53 mutation possible, MGMT methylated, EGFR amplification, MET amplification, CDKN2A/B deletion, + 7/−10	Dexamethasone, levetiracetam	70%
11	68	female	GBM, IDH-wildtype, ATRX-wildtype, MGMT methylated, PDGFRA amplification, MET amplification, CDKN2A/B deletion	Dexamethasone, amitriptyline	80%
12	75	male	GBM, IDH-wildtype, ATRX-wildtype, MGMT methylated	Dexamethasone, levetiracetam	40%

ATRX = alpha-thalassemia/mental retardation syndrome X-linked; CDK4 = cyclin-dependent kinase 4; CDKN2A/B = cyclin-dependent kinase inhibitor 2 A/B; ECOG = Eastern Cooperative Oncology Group; EGFR = epidermal growth factor receptor; GBM = glioblastoma; H3K27me3 = trimethylation of histone H3 at lysine 27; IDH = isocitrate dehydrogenase; MDM2 = mouse double minute 2 homolog; MET = MET proto-oncogene; MGMT = O6-methylguanine-DNA methyltransferase; GFRA = glial cell line-derived neurotrophic factor family receptor alpha; PDGFRA = platelet-derived growth factor receptor alpha; PTEN = phosphatase and tensin homolog; RB1 = retinoblastoma 1; TP53 = tumor protein p53; +7/−10 = combined whole-chromosome 7 gain and chromosome 10 loss

^a^No patient underwent neurosurgical treatment prior to the BOLD MRI examination

### BOLD MRI, anatomical MRI and DSC MR perfusion data

BOLD MRI data were acquired on a 3 Tesla Skyra VE11 (Siemens, Erlangen, Germany) scanner using a 32-channel head coil. BOLD MRI data acquisition was prescribed along the AC-PC line plus 20° anticlockwise on a sagittal localizer and performed using T2*-weighted echo-planar imaging (EPI) sequences with the following parameters: repetition time (TR) = 1800 ms, echo time (TE) = 30 ms, slice thickness = 2.5 mm, 50 slices in interleaved order, field of view (FOV) = 220 × 220 mm^2^, flip angle (FA) = 80°, in-plane resolution = 2.5 × 2.5 mm (voxel size 2.5 × 2.5 × 2.5 mm^3^), 160 measurements, acquisition time (TA) = 5:00 min.

High-resolution three-dimensional T1w anatomical images were acquired in the same orientation as the BOLD MRI sequence and within the same imaging session to enable accurate co-registration. Acquisition parameters were as follows: TR = 2200 ms, TE = 5.14 ms, 176 slices per slab with a thickness of 1 mm, FOV = 230 × 230 mm^2^, in-plane resolution = 0.8 × 0.8 mm (voxel size 0.8 × 0.8 × 1 mm^3^), FA = 8°, TA = 8:14 min.

T2-weighted (T2w), fluid-attenuated inversion recovery (FLAIR), post-contrast T1-weighted (T1pc) sequences, and DSC MR perfusion data were obtained from clinical MRI scans.

### Respiratory protocol

During BOLD MRI data acquisition, a custom-built computer-controlled gas blender (RespirAct™ Gen 4, Thornhill Medical, Toronto, Canada) was employed to precisely and independently control the end-tidal partial pressure of O_2_ (P_ET_O_2_) and CO_2_ (P_ET_CO_2_) using a prospective gas targeting algorithm as previously described [23]. A double step hypoxic stimulus protocol was induced starting with a 60 s baseline of P_ET_O_2_ 95 mmHg (normoxia), followed by two 60 s hypoxic steps with P_ET_O_2_ reduced to 40 mmHg (hypoxia), interleaved by a return to normoxia for 40 s and finally a return to baseline for 60 s.

### MRI and respiratory data processing

Statistical Parametric Mapping (SPM12, Wellcome Trust Centre for Neuroimaging, London; http://www.fil.ion.ucl.ac.uk/spm) running on MATLAB was used for data preprocessing. BOLD and T1w images were converted from DICOM to NIfTI format. The BOLD images were slice-time corrected, rigidly realigned for head motion correction, and co-registered to the T1w image. Tissue segmentation of the T1w image yielded gray matter (GM), white matter (WM), and cerebrospinal fluid (CSF) probability maps in native space, which were resliced into BOLD space and used as brain parenchyma masks. The same segmentation step also generated a subject-specific forward deformation field. This deformation field represents a voxel-wise spatial transformation mapping native T1w space into Montreal Neurological Institute (MNI) space and was subsequently applied to warp the co-registered BOLD images into MNI space. Normalized BOLD images were spatially smoothed with an isotropic Gaussian kernel (full width at half maximum (FWHM) = 6 mm). The masked BOLD signal time series were low-pass filtered (cutoff frequency = 0.125 Hz) and smoothed using robust LOESS (window size = 16 volumes, 28.8 s).

**Fig. 1:**
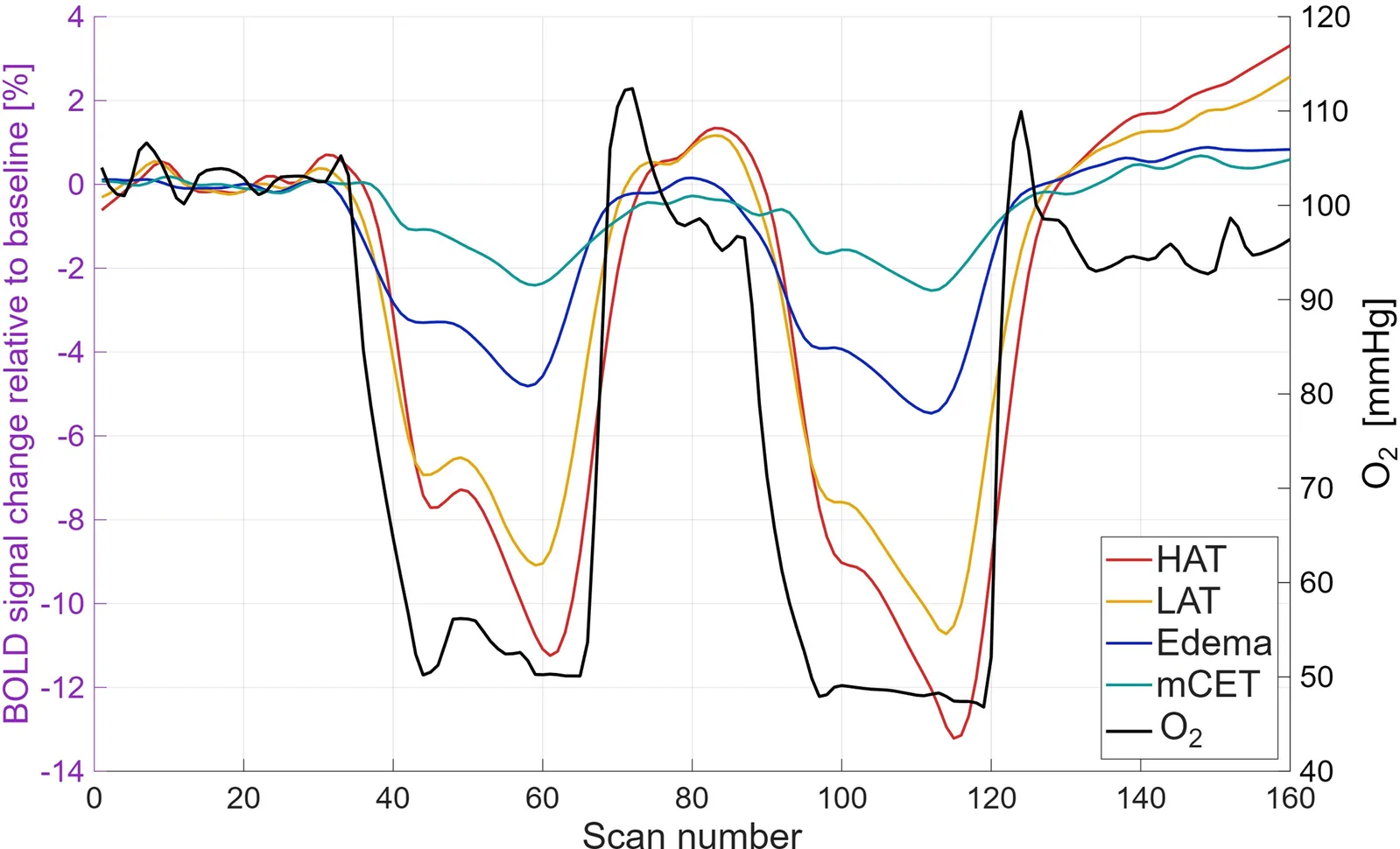
Exemplary end-tidal partial pressure of O_2_ (P_ET_O_2_) time course and the corresponding globally shifted BOLD signal time courses of each volume of interest. HAT = high angiogenic tumor, LAT = low angiogenic tumor, mCET = mirrored contrast-enhancing tissue

The P_ET_O₂ time series was resampled to match the BOLD time series using piecewise cubic Hermite interpolation. A global hemodynamic delay was estimated by cross-correlating the brain-averaged BOLD signal with the interpolated P_ET_O₂ time series and used to temporally align it. A voxel-wise oxygen reference trace was then generated by applying an iteratively estimated, voxel-specific delay to this globally aligned trace, as recently proposed [3]. Voxel-wise BOLD signal changes were subsequently calculated during the steady-state plateau of the first hypoxic stimulus block, normalized to the achieved end-tidal oxygen step amplitude. The double-stimulus design performed in this study was based on earlier methodological approaches [1]; however, as the steady-state analysis of the first stimulus block was recently identified as the superior approach due to avoidance of carryover effects on transition dynamics [3], only the first stimulus block was analyzed accordingly in the present study. Figure 1 shows an exemplary illustration of the P_ET_O₂ time series and corresponding BOLD signal time courses.

### Tumor and brain tissue segmentation

Tumor segmentation was based on T2w, FLAIR, T1pc, and DSC MR perfusion data. T2w, FLAIR and T1pc images were co-registered to the native T1w space and subsequently normalized to MNI space using the patient-specific deformation fields from the preprocessing step. Automated segmentation was performed using the Oncohabitats framework [24–26], which generated masks for contrast-enhancing tumor, necrosis, and peritumoral edema from T2w, FLAIR, and T1pc images. Oncohabitats additionally uses DSC MR perfusion to subdivide the tumor tissue and surrounding edema into four vascular habitats: the high angiogenic tumor (HAT), the low angiogenic tumor (LAT), the potentially infiltrated peripheral edema (IPE), and the vasogenic peripheral edema (VPE) [24–26]. The IPE habitat was not included as a separate VOI, as its spatial definition requires direct adjacency to the contrast-enhancing tumor [26]; however, we hypothesized that hypoxia-targeted BOLD MRI hotspots reflecting infiltrated tumor tissue might also occur without direct spatial adjacency. The VPE habitat was likewise not analyzed as a separate VOI; instead, we used the peritumoral edema as segmented by Oncohabitats as a whole (encompassing both IPE and VPE), without further subdividing it into these two vascular sub-compartments. We therefore selected the following volumes of interest (VOIs) for BOLD signal intensity and heterogeneity assessment: the HAT and LAT, corresponding directly to the high and low angiogenic tumor habitats as defined by the Oncohabitats framework; the peritumoral edema, corresponding to the Oncohabitats-defined edema mask; and the contralateral mirror region of the contrast-enhancing tissue (mCET). Unlike the other three VOIs, mCET is not itself an Oncohabitats-defined habitat, but was instead derived by geometric reflection of the Oncohabitats-defined contrast-enhancing lesion mask along the sagittal midline, serving as an intra-individual reference of healthy-appearing tissue. To provide a quantitative basis for the tissue composition of each VOI, GM and WM fractions were derived from the SPM12 tissue segmentation performed during preprocessing and descriptively reported. As direct segmentation might be unreliable within pathological tissue, GM/WM fractions for HAT, LAT, and peritumoral edema were estimated using the mirrored, presumed-healthy contralateral counterpart of each region.

### BOLD signal intensity and heterogeneity maps

For visual inspection, hypoxia-targeted BOLD signal changes and heterogeneity maps were displayed voxel-wise as overlays on the T1pc images. Heterogeneity maps were generated by computing, for each voxel, the spatial standard deviation (SD) of the voxel-wise BOLD signal change values across the 27 voxels within its 3 × 3 × 3 voxel neighborhood. Relative cerebral blood volume (rCBV) maps, generated by the Oncohabitats DSC MR perfusion processing pipeline [24, 26, 27] were resliced to the patient’s native T1w space using SPM12, and also displayed.

### Analysis of BOLD signal magnitude and heterogeneity

To quantitatively characterize voxel-wise signal distributions within the VOIs, kernel density estimation (KDE) was applied. KDE was preferred over conventional histogram-based approaches as it provides a continuous, bin-independent representation of the underlying probability density function, avoiding the arbitrary influence of bin width and placement on distributional shape. KDE was performed using a Gaussian kernel with bandwidth determined by Silverman’s rule of thumb [28]. A single bandwidth was computed per patient from the pooled voxel values across all regions to avoid VOI-specific smoothing effects and to enable direct comparison of density estimates. Differential entropy was computed from the estimated densities using a discrete approximation, quantifying distributional heterogeneity independently of the mean. Descriptive statistics (mean and interquartile range (IQR)) were calculated from voxel intensities within each VOI. As voxel-wise BOLD signal intensities within each VOI and patient were predominantly non-normally distributed (see Statistical Analysis), median and IQR were used to characterize the magnitude and heterogeneity of the BOLD signal, respectively with entropy used as an additional heterogeneity metric.

### Statistical analysis

Statistical analysis was performed with MATLAB and SPSS Statistics (IBM Corp. Released 2021. IBM SPSS1 Statistics for Windows, Version 28.0. Armonk, NY, USA: IBM Corp).

First, normality of voxel intensities within each VOI and patient was assessed using the Lilliefors test. Due to the predominantly non-normally distributed data, median was used as the primary descriptive measure of magnitude and IQR together with entropy was used as heterogeneity metrics for further group comparisons. Due to the small sample size, a Friedman test with Dunn post-hoc tests was used to assess differences between median, IQR, and entropy of each VOI. Resulting uncorrected p-values were subsequently corrected for multiple comparisons within each metric using the Benjamini-Hochberg false discovery rate (FDR) procedure. The significance level was set at *p* < 0.05.

Effect sizes were calculated for the overall Friedman test (Kendall’s W) and for pairwise post-hoc comparisons (rank-biserial correlation r). Effect sizes were interpreted according to conventional thresholds adapted from Cohen’s benchmarks, with W or *r* ≥ 0.1, ≥ 0.3, and ≥ 0.5 indicating small, moderate, and large effects, respectively [29]. For each pairwise VOI comparison, the Hodges-Lehmann estimator and corresponding 95% confidence interval for the median paired difference were additionally calculated using the Wilcoxon signed-rank framework, to complement the effect size and significance estimates obtained from the Friedman/Dunn analysis.

## Results

### Patients

In total, 12 patients (3 female, 9 male) with isocitrate dehydrogenase-wildtype glioblastoma were included in the study. The mean age was 61.4 ± 8.1 years. The mean interval between BOLD MRI and DSC MR perfusion data acquisition was 3.1 ± 4.7 days.

### BOLD signal change and heterogeneity maps

**Fig. 2:**
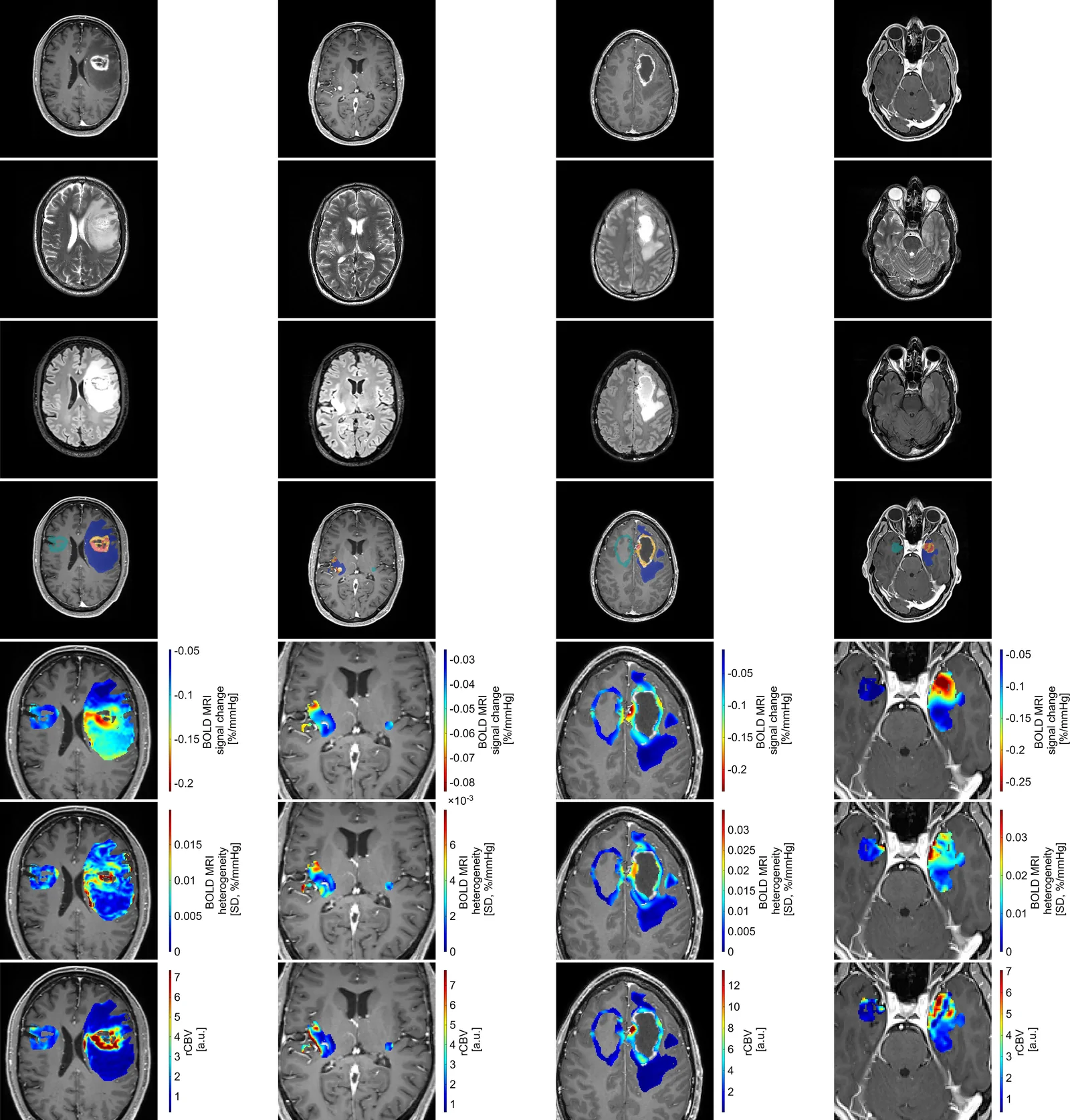
MRI data of four patients with glioblastoma. First row: T1-weighted post-contrast; second row: T2-weighted; third row: fluid-attenuated inversion recovery (FLAIR); fourth row: depiction of the volumes of interest. Red = high angiogenic tumor, orange = low angiogenic tumor, blue = edema, green = mirrored contrast-enhancing tissue; fifth row: BOLD signal change; sixth row: BOLD signal heterogeneity; seventh row: relative cerebral blood volume (rCBV)

Exemplary BOLD signal change and heterogeneity maps, as well as the corresponding anatomical images and rCBV maps, are presented in Fig. 2. The maps of the other patients are provided as supplementary Figure S1. In most cases, the HAT demonstrated not only more pronounced BOLD signal changes than the other VOIs, but also greater signal heterogeneity. In edema and in the mCET, smaller and more homogeneous BOLD signal changes were observed. LAT showed intermediate values.

### Overview of BOLD signal change distributions across VOIs

**Fig. 3:**
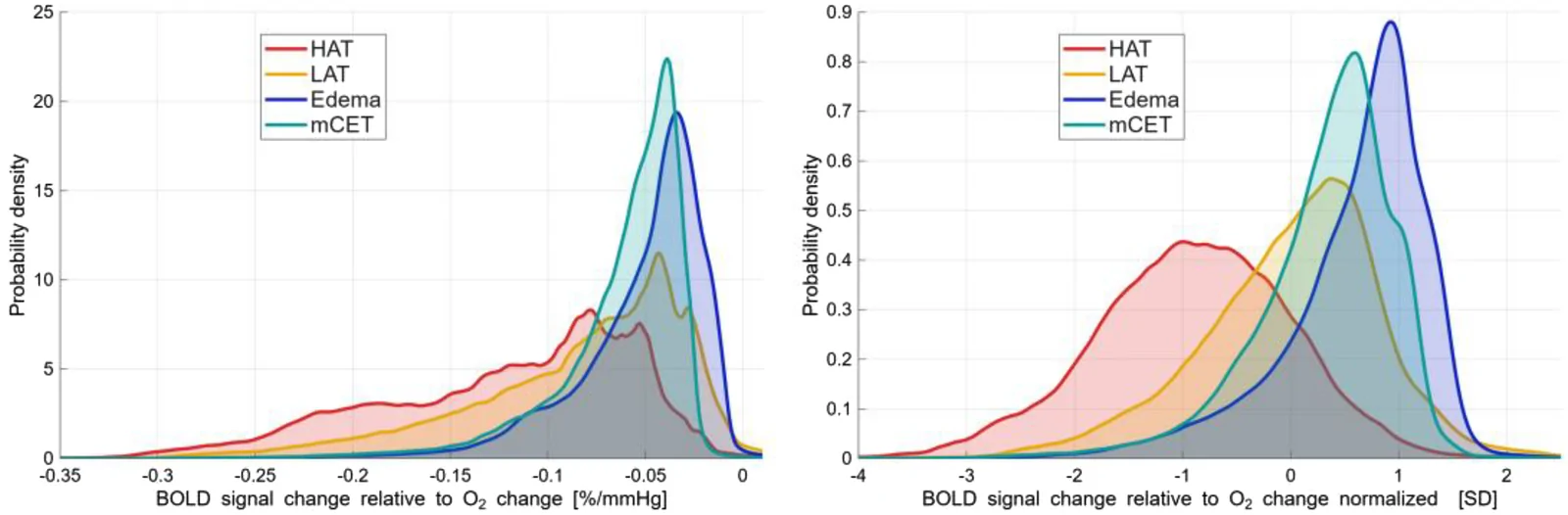
Group-averaged kernel density estimation maps of the BOLD signal change in the volumes of interest high angiogenic tumor (HAT), low angiogenic tumor (LAT), edema and mirrored contrast-enhancing tissue (mCET). BOLD signal values are presented in the original units (%/mmHg) and in standard deviation (SD) units, as patients show different BOLD signal ranges

Group-averaged KDE plots are shown in Fig. 3, presented both in the original units (%/mmHg) and as patient-pooled, SD-normalized units. The SD-normalized representation was chosen in addition to the original-unit plot because inter-patient variability in absolute BOLD signal scale can obscure shape-level differences between VOIs (e.g., relative spread, skewness) when distributions from multiple patients are pooled; SD normalization removes this scale-dependent variability and thereby facilitates visual comparison of distribution shape across VOIs. Of note, the entropy values reported throughout this manuscript are calculated on each patient’s raw, non-normalized voxel-wise BOLD signal values and are therefore unaffected by this normalization, which was applied for visualization purposes only.

The BOLD signal appears more heterogeneously distributed, particularly in the HAT and to a lesser extent in the LAT, compared with edema and mCET. Furthermore, it is evident that the hypoxia-targeted BOLD signal changes are greater in the HAT than the other VOIs, although substantial inter-VOI overlap was observed.

### Tissue composition of VOIs

The estimated ratio of median GM: WM values was 1.61 in HAT, 1.20 in LAT, 1.20 in mCET, and 0.82 in peritumoral edema, indicating a higher WM content in the edema VOI.

### Comparison of BOLD signal change

**Fig. 4:**
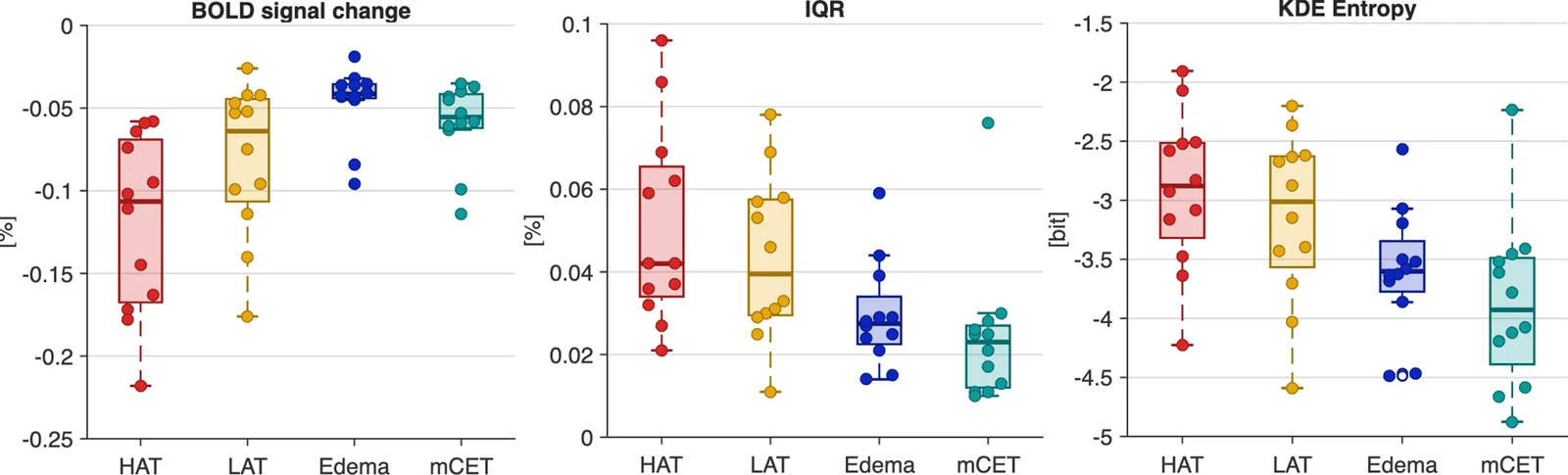
Boxplots showing distribution of patients‘ median BOLD signal change, interquartile range (IQR) and entropy across the volumes of interest: high angiogenic tumor (HAT), low angiogenic tumor (LAT), edema and mirrored contrast-enhancing tissue (mCET)

Median BOLD signal change values differed significantly across all VOIs, with a large effect size (Kendall’s W = 0.778, *p* < 0.001; see Tables 2 and 3; Fig. 4). The HAT exhibited the largest magnitude of BOLD signal change, which was significantly greater than in the LAT (*r* = 0.365, FDR-adjusted *p* = 0.017), edema (*r* = 0.730, FDR-adjusted *p* < 0.001), and mCET (*r* = 0.548, FDR-adjusted *p* < 0.001). The LAT showed the second most pronounced BOLD signal change, with a significantly larger decrease compared to edema (*r* = 0.365, FDR-adjusted *p* = 0.017).

**Table 2:** Comparison of BOLD signal change and heterogeneity metrics across volumes of interest

	BOLD signal change [%/mmHg] Median (IQR across patients)		IQR^a^ [%/mmHg] Median (IQR across patients)	Entropy [bit] Median (IQR across patients)
HAT		-0.107 (0.103)	0.042 (0.035)	-2.878 (0.886)
LAT		-0.064 (0.067)	0.039 (0.028)	-3.013 (1.013)
Edema		-0.042 (0.009)	0.028 (0.014)	-3.602 (0.549)
mCET		-0.056 (0.022)	0.023 (0.016)	-3.929 (1.017)

HAT = high angiogenic tumor, LAT = low angiogenic tumor, mCET = mirrored contrast-enhancing tissue, IQR = interquartile range. Values are presented as median (IQR) across the 12 patients

^a^Note that the column 'IQR [%/mmHg]' itself reports a per-patient heterogeneity metric (the IQR of voxel-wise BOLD intensities within each VOI); the bracketed value in this column therefore represents the IQR (across patients) of these per-patient IQR values, i.e., a second-order dispersion measure

**Table 3:** Comparison of effect sizes of differences in BOLD signal change and heterogeneity metrics across volumes of interest

	BOLD signal change [%]			IQR [%]			Entropy [bit]		
	Median difference (HL estimate, 95% CI)	Rank-biserial *r*	FDR-adjusted *p*	Median difference (HL estimate, 95% CI)	Rank-biserial *r*	FDR-adjusted *p*	Median difference (HL estimate, 95% CI)	Rank-biserial *r*	FDR-adjusted *p*
HAT-LAT	0.039 (0.028, 0.050)	0.365	0.017	0.008 (-0.003, 0.017)	0.068	0.635	0.180 (-0.017, 0.499)	0.137	0.412
HAT-Edema	0.074 (0.042, 0.101)	0.730	< 0.001	0.016 (0.007, 0.037)	0.411	0.008	0.685 (0.382, 0.987)	0.479	0.003
HAT-mCET	0.055 (0.033, 0.089)	0.548	< 0.001	0.025 (0.011, 0.041)	0.525	0.002	1.004 (0.450, 1.453)	0.571	< 0.001
LAT-Edema	0.034 (0.008, 0.054)	0.365	0.017	0.013 (0.005, 0.022)	0.342	0.027	0.453 (0.196, 0.725)	0.342	0.027
LAT-mCET	0.014 (-0.002, 0.041)	0.183	0.206	0.018 (0.005, 0.033)	0.456	0.006	0.751 (0.286, 1.138)	0.434	0.005
Edema-mCET	-0.012 (-0.023, 0.000)	-0.183	0.206	0.006 (-0.003, 0.016)	0.114	0.515	0.302 (-0.152, 0.712)	0.091	0.527

CI = confidence interval; FDR = false discovery rate; HAT = high angiogenic tumor, HL = Hodges-Lehmann estimate; LAT = low angiogenic tumor, mCET = mirrored contrast-enhancing tissue, IQR = interquartile range. Values are presented as median [IQR] across the 12 patients. Note that the Friedman/Dunn test and the Wilcoxon-based Hodges-Lehmann estimator operate on different internal rank/value conventions; signs were therefore harmonized post hoc so that both measures consistently indicate the same direction of effect within each pairwise comparison, with positive values indicating a stronger effect (greater BOLD signal decrease, IQR, or entropy) in the first-named region of each comparison

### Comparison of BOLD signal heterogeneity

Median IQR of the BOLD signal distribution differed significantly between the VOIs with large effect size (Kendall’s W = 0.525, *p* < 0.001; see Tables 2 and 3; Fig. 4). The HAT demonstrated the highest BOLD signal heterogeneity with a significantly increased IQR compared to both the edema (*r* = 0.411, FDR-adjusted *p* = 0.008) and the mCET (*r* = 0.525, FDR-adjusted *p* = 0.002). LAT likewise exhibited significantly higher IQR values than edema (*r* = 0.342, FDR-adjusted *p* = 0.027) and mCET (*r* = 0.456, FDR-adjusted *p* = 0.006).

Entropy analysis revealed consistent findings, with significant differences across all VOIs with large effect size (Kendall’s W = 0.592, *p* < 0.001; Tables 2 and 3; Fig. 4). The highest entropy was observed in the HAT, which was significantly greater than in edema (*r* = 0.479, FDR-adjusted *p* = 0.003) and mCET (*r* = 0.571, FDR-adjusted *p* < 0.001). LAT showed significantly increased entropy compared to edema (*r* = 0.342, FDR-adjusted *p* = 0.027) and mCET (*r* = 0.434, FDR-adjusted *p* = 0.005).

## Discussion

Hypoxia-targeted BOLD MRI represents a promising technique for the characterization of tumor physiology in glioblastoma [1–3, 14]. It is known that hypoxia-induced stimuli lead to a more pronounced BOLD signal decrease in brain tumors compared to healthy tissue [2], which might be explained by differences in vascularization and hypoxic tumor environment.

Given the highly heterogeneous appearance of glioblastoma, which is also reflected in other MRI-based approaches [17, 18, 30–36], the aim of this pilot study was not only to assess hypoxia-targeted BOLD signal magnitude but also to characterize and investigate hypoxia-targeted BOLD signal heterogeneity.

Regarding signal magnitude, BOLD signal decreases were significantly more pronounced in highly angiogenic tissue verified by the Oncohabitats framework and thus presumed to represent more aggressive tumor components [25], compared with LAT, edema, and the mirrored control region – an overall pattern reflected in a large effect size – although substantial overlap between the VOIs was observed. These findings are consistent with our expectations [1, 2]. Interestingly, the BOLD signal decrease in the contralateral control region was more pronounced than in peritumoral edema, without reaching statistically significance. This may be explained by the preferential spread of peritumoral edema along WM tracts [37, 38], resulting in a higher proportion of WM within the peritumoral edema ROI compared with the mirrored control ROI – consistent with the estimated GM: WM ratio being lower in edema than in the mirrored control region. Since BOLD signal changes are typically smaller in WM than in GM [5] – owing to lower vascular density, cerebral blood flow, and blood volume [39, 40]–this tissue composition difference may account for the comparatively attenuated BOLD response observed in the edema region.

To assess heterogeneity, IQR and entropy were used as quantitative metrics. In this context, we understood signal heterogeneity as the extent to which hypoxia-targeted BOLD responses vary across voxels within a given tumor subregion – a pattern that may indicate the co-existence of different physiological or microstructural compartments (e.g., regions with divergent vascularization and shunting, variable oxygen extraction fraction (OEF), or a mixture of necrotic and viable tissue) [8, 17, 18, 20, 41, 42] rather than a spatially uniform tissue-level response. IQR reflects the overall dispersion of voxel-wise signal values, whereas KDE-derived entropy additionally captures the shape and complexity of the entire distribution, including multimodal or skewed patterns that dispersion alone may not reveal. Both measures demonstrated significantly higher signal heterogeneity in the HAT compared with edema and the contralateral control region, and heterogeneity was non-significantly increased compared with LAT, consistent with the large overall effect sizes for both metrics. No significant differences in heterogeneity were observed between edema and the control region. In the patient maps, we observed a qualitative, partial visual correspondence between regions of high BOLD signal heterogeneity and highly angiogenic tumor components identified by the Oncohabitats framework [25]; this observation was not formally quantified and should be regarded as preliminary.

The findings of this pilot study suggest that the assessment of hypoxia-targeted BOLD signal heterogeneity may provide clinically relevant information. Previous studies have associated MRI signal heterogeneity with reduced overall survival [18]. Signal heterogeneity may reflect differences in tumor cell differentiation, the distribution of intratumoral vessels, and the metabolic status of tumor cells [32]. By providing insights into metabolic alterations [2], hypoxia-targeted BOLD MRI may contribute to the characterization of intratumoral metabolic heterogeneity.

We acknowledge that DSC MR perfusion and hypoxia-targeted BOLD are not fully independent contrasts, because both are sensitive to vascular architecture, blood volume, and deoxyhemoglobin-related susceptibility effects [43]. Therefore, perfusion-derived Oncohabitats HAT and LAT subregions should not be regarded as a fully external reference standard. However, DSC MR perfusion and hypoxia-targeted BOLD offer differences in tracer kinetics and biophysical origin. DSC MR perfusion is governed by susceptibility bolus properties and vascular compartmentalization [44], whereas BOLD-based oxygenation measures reflect endogenous deoxyhemoglobin content, which is jointly determined by blood volume and local oxygen extraction, rather than CBV alone [42]. In glioma, these oxygenation-related parameters are spatially heterogeneous, and although the OEF tends to be lower in brain tumors overall compared to healthy tissue [45], higher-grade or more aggressive tumors have been reported to show higher focal OEF abnormalities [41, 46, 47]. In addition, lower pH reduces hemoglobin oxygen affinity via the Bohr effect [11], making it physiologically plausible that acidic tumor regions could show enhanced deoxyhemoglobin accumulation during a hypoxic challenge. Because vascular deoxygenation and tissue hypoxia can be spatially discordant in glioma [48], we interpret the observed BOLD heterogeneity as overlapping, but complementary information relative to the DSC MR perfusion-based labels.

This study has limitations. First, the sample size was limited, which carries an inherent risk of underpowering the analysis. To quantify this risk, we report effect size estimates alongside p-values throughout the Results. With *n* = 12 patients and the related-samples framework used here, our study had reasonable power to detect large effects (*r* ≥ 0.5, as observed for e.g. HAT vs. mCET or HAT vs. edema across all three metrics) but limited power to reliably detect small-to-moderate effects (*r* ≈ 0.1–0.3). We recommend that future confirmatory studies use the effect sizes reported here to inform formal a priori power calculations. Although no study participant received neurosurgical therapy prior to BOLD MRI examination, most patients received steroid administration, which may have influenced the signal in edema to varying degrees, and which the small sample size did not allow us to control for statistically.

Second, the quantitative heterogeneity analyses were performed using VOI segmentation derived from a deep-learning-based approach based on morphological MRI and DSC MR perfusion rather than from histopathological ground truth [49–54]. Third, this work focused on contrast-enhancing tumor components of varying vascularity. A clinically relevant question remains the detection of non-contrast-enhancing infiltrative tumor tissue [55, 56]. However, due to the lack of an available reliable reference standard for infiltration beyond contrast enhancement, this aspect could not be addressed in the present study. Fourth, all data were acquired on a single 3T scanner at a single center, and the applied spatial smoothing kernel during preprocessing directly influences the absolute magnitude of the voxel-wise heterogeneity metrics (IQR, entropy); a formal sensitivity analysis across different kernel sizes and multiple scanners/centers was not performed in this pilot study and represents an important direction for future work. Additionally, the temporal low-pass filter and LOESS smoother applied to voxel-wise BOLD time series during preprocessing – adopted from prior studies about hypoxia-targeted BOLD MRI to reduce physiological and scanner noise [2] – could in principle also attenuate genuine biological variability in the temporal response, thereby indirectly underestimating the true spatial heterogeneity captured by IQR and entropy. The sensitivity of these heterogeneity metrics to the choice of temporal smoothing parameters was not formally assessed in this pilot study and represents an important direction for future work. Fifth, DSC MR perfusion imaging, used for Oncohabitats-based VOI segmentation, was permitted to have been acquired up to two weeks before or after the BOLD MRI examination. Given the potentially rapid growth and evolving vascular architecture of glioblastoma, this interval may have introduced a degree of spatial or physiological mismatch between the perfusion-derived VOI boundaries and the tumor state at the time of BOLD acquisition, representing a further potential source of error or confounding not accounted for in the present analysis.

Future studies should validate the findings of this pilot study in larger cohorts and against more robust reference standards. An important topic for further investigation is the potential relationship between tumor heterogeneity and patient prognosis, including overall survival [18].

Hypoxia-targeted BOLD MRI is a relatively novel approach in glioblastoma imaging that requires further clinical validation and methodological refinement. Ultimately, the aim is to identify infiltrative tumor tissue that is not detectable with conventional imaging techniques and to differentiate it from treatment-related changes. This study indicates that the assessment of BOLD signal heterogeneity may provide valuable insights in this context.

## Conclusion

In this pilot study, hypoxia-targeted BOLD MRI demonstrated not only significantly greater BOLD signal changes in highly angiogenic tumor subregions, as identified by an automated segmentation framework based on morphological MRI and DSC MR perfusion, but also increased intratumor heterogeneity. These findings require validation in larger cohorts; however, they suggest that heterogeneity analysis may represent a valuable complementary metric in the assessment of hypoxia-targeted BOLD MRI.

## Supplementary Information

Below is the link to the electronic supplementary material.

Supplementary Material 1

## Data Availability

The datasets analyzed during the current study are available from the corresponding author on reasonable request.

## Declarations

### AI Declaration Statement

During the preparation of this work the authors used Claude (Anthropic) in order to improve the readability and language of the manuscript and for assistance with MATLAB code generation. After using this tool/service, the authors reviewed and edited the content as needed and take full responsibility for the content of the published article.
