# Supplementary material for: Hypoxia-targeted BOLD MRI signal heterogeneity as a complementary metric for glioblastoma characterization: a pilot study
Source: J Neurooncol. 2026 Aug 20;179(2):47. doi: 10.1007/s11060-026-05762-6 (PMC13493451; doi:10.1007/s11060-026-05762-6)
Supplement: Supplementary file 1 [file 11060_2026_5762_MOESM1_ESM.pdf]

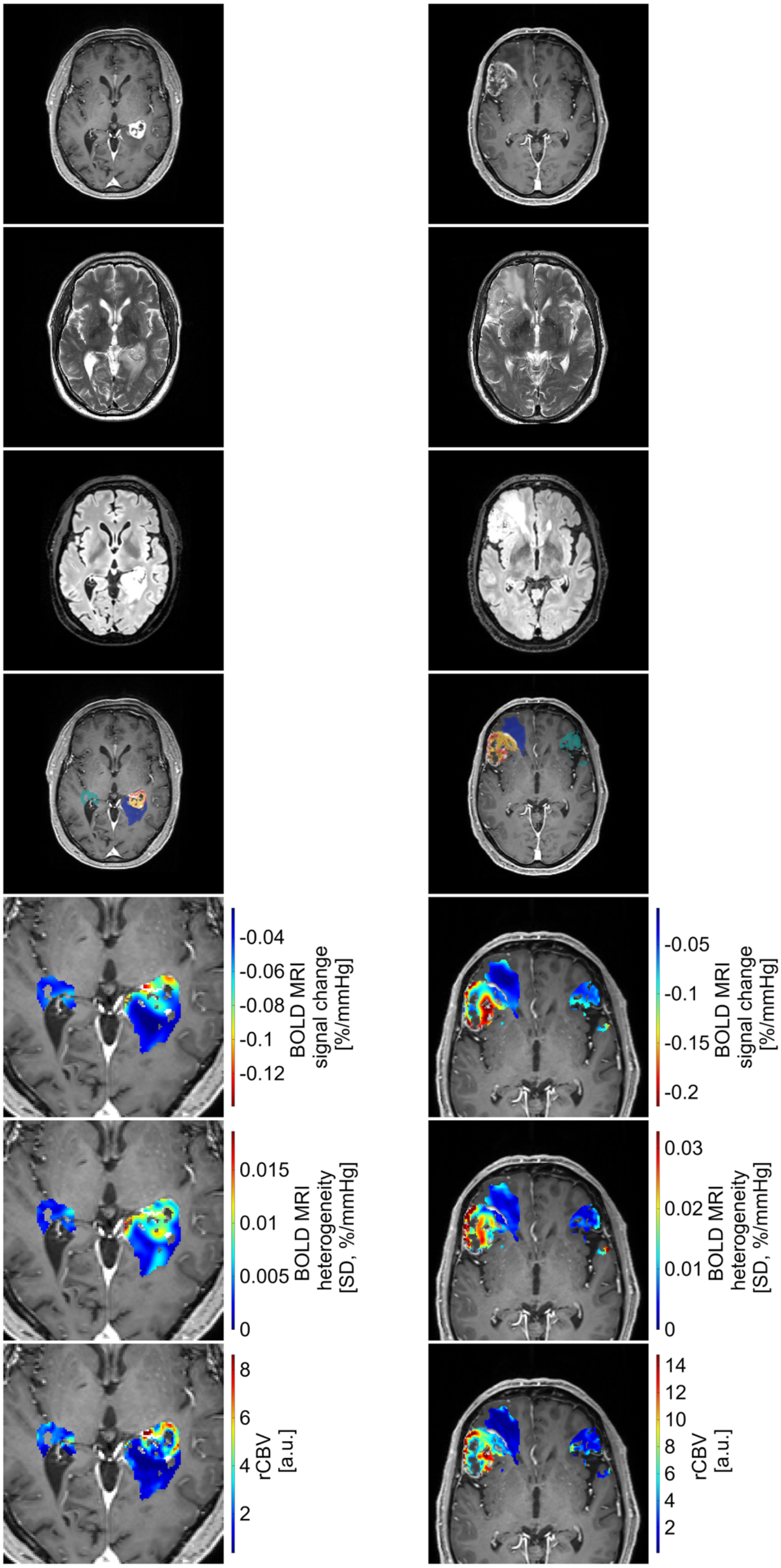
-0.04
-0.06
-0.08
-0.1
-0.12
BOLD MRI signal change [%/mmHg]
0.015
0.01
0.005
0
BOLD MRI heterogeneity [SD, %/mmHg]
8
6
4
2
rCBV [a.u.]
-0.05
-0.1
-0.15
-0.2
BOLD MRI signal change [%/mmHg]
0.03
0.02
0.01
0
BOLD MRI heterogeneity [SD, %/mmHg]
14
12
10
8
6
4
2
rCBV [a.u.]

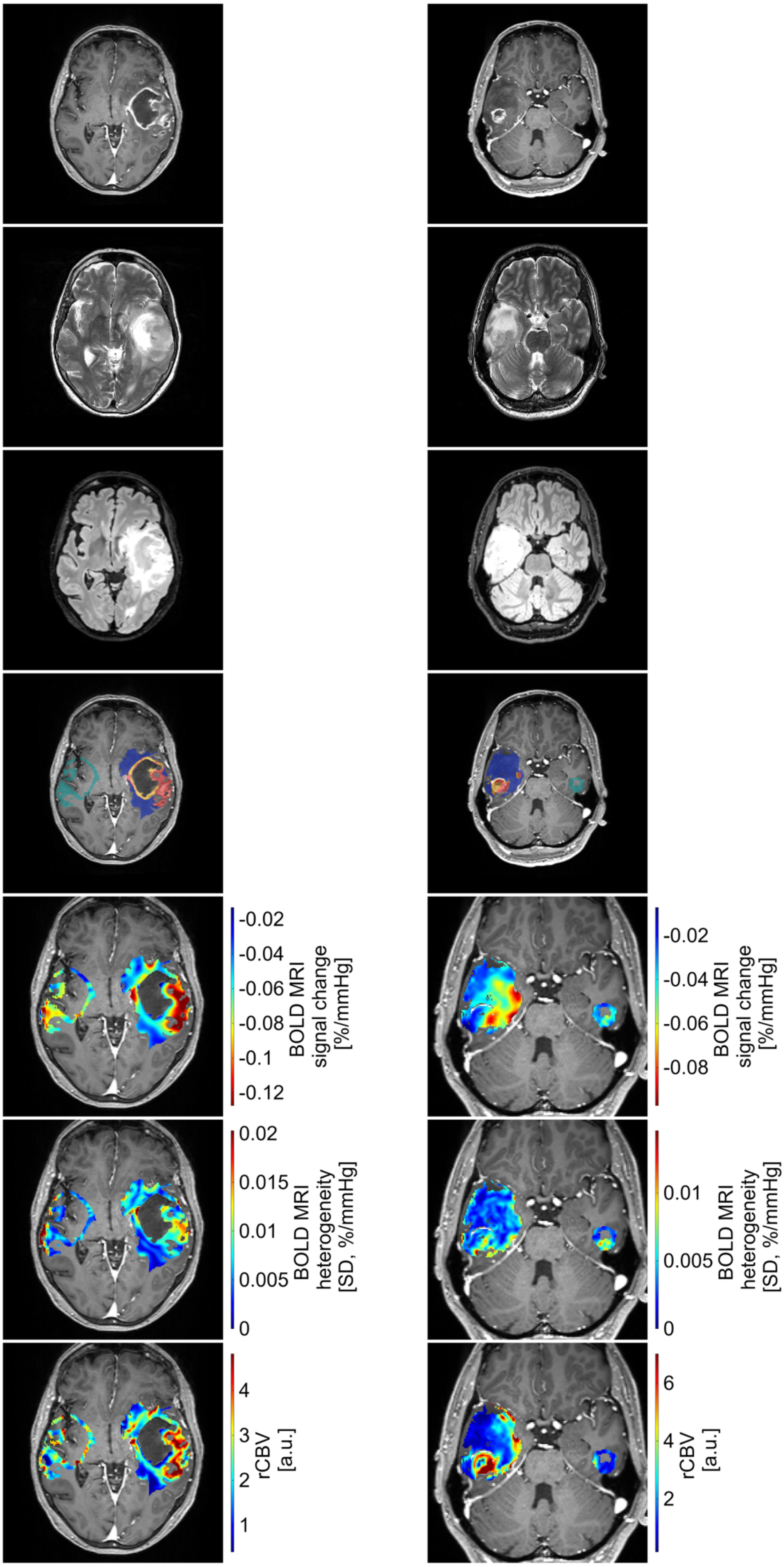
-0.02
-0.04
-0.06
-0.08
-0.1
-0.12
BOLD MRI
signal change
[%/mmHg]
0.02
0.015
0.01
0.005
0
BOLD MRI
heterogeneity
[SD, %/mmHg]
4
3
2
1
rCBV
[a.u.]
-0.02
-0.04
-0.06
-0.08
BOLD MRI
signal change
[%/mmHg]
0.01
0.005
0
BOLD MRI
heterogeneity
[SD, %/mmHg]
6
4
2
rCBV
[a.u.]

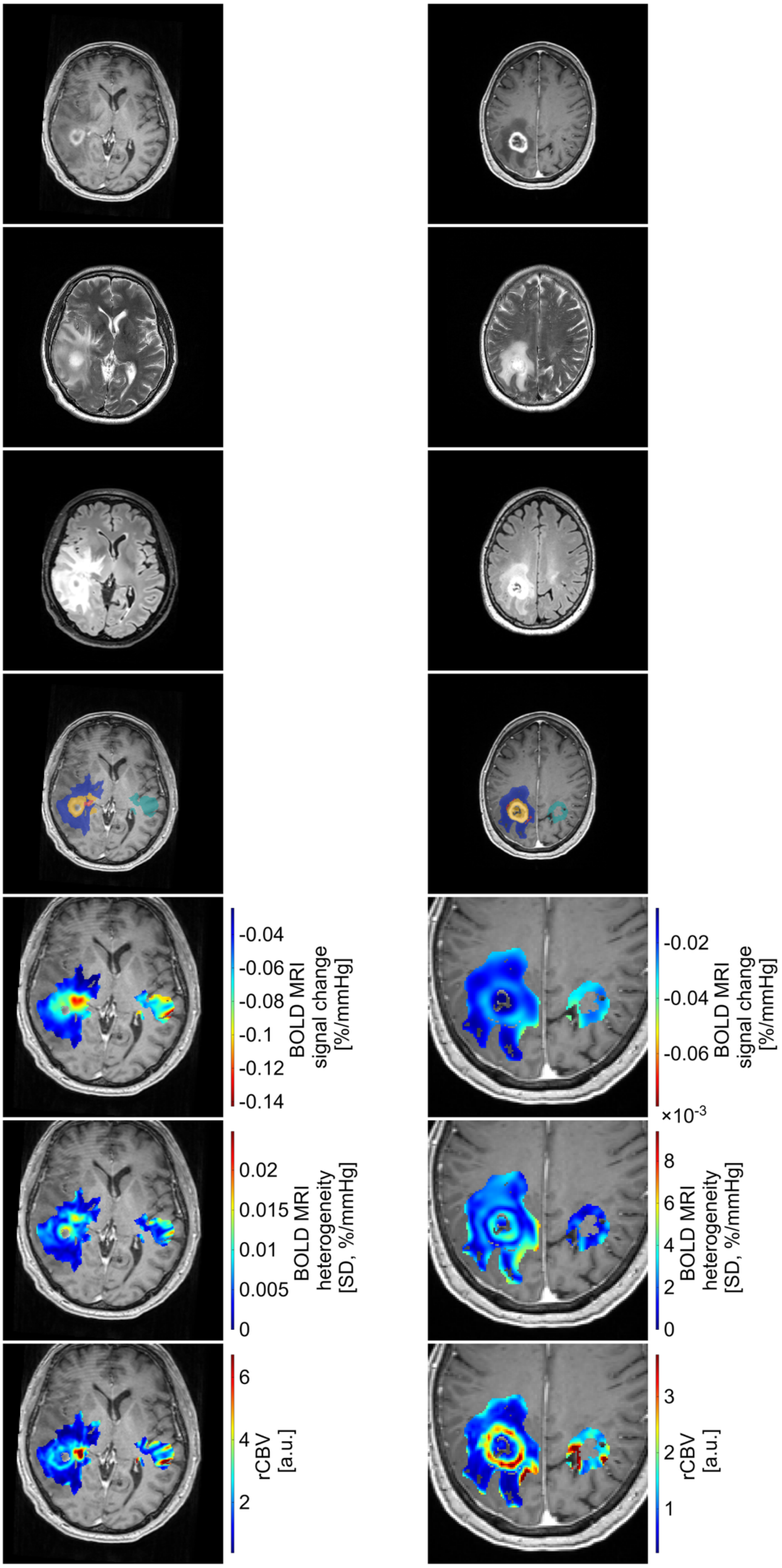
-0.04
-0.06
-0.08
-0.1
-0.12
-0.14
BOLD MRI
signal change
[%/mmHg]
0.02
0.015
0.01
0.005
0
BOLD MRI
heterogeneity
[SD, %/mmHg]
6
4
2
rCBV
[a.u.]
-0.02
-0.04
-0.06
BOLD MRI
signal change
[%/mmHg]
×10-3
8
6
4
2
0
BOLD MRI
heterogeneity
[SD, %/mmHg]
3
2
1
rCBV
[a.u.]

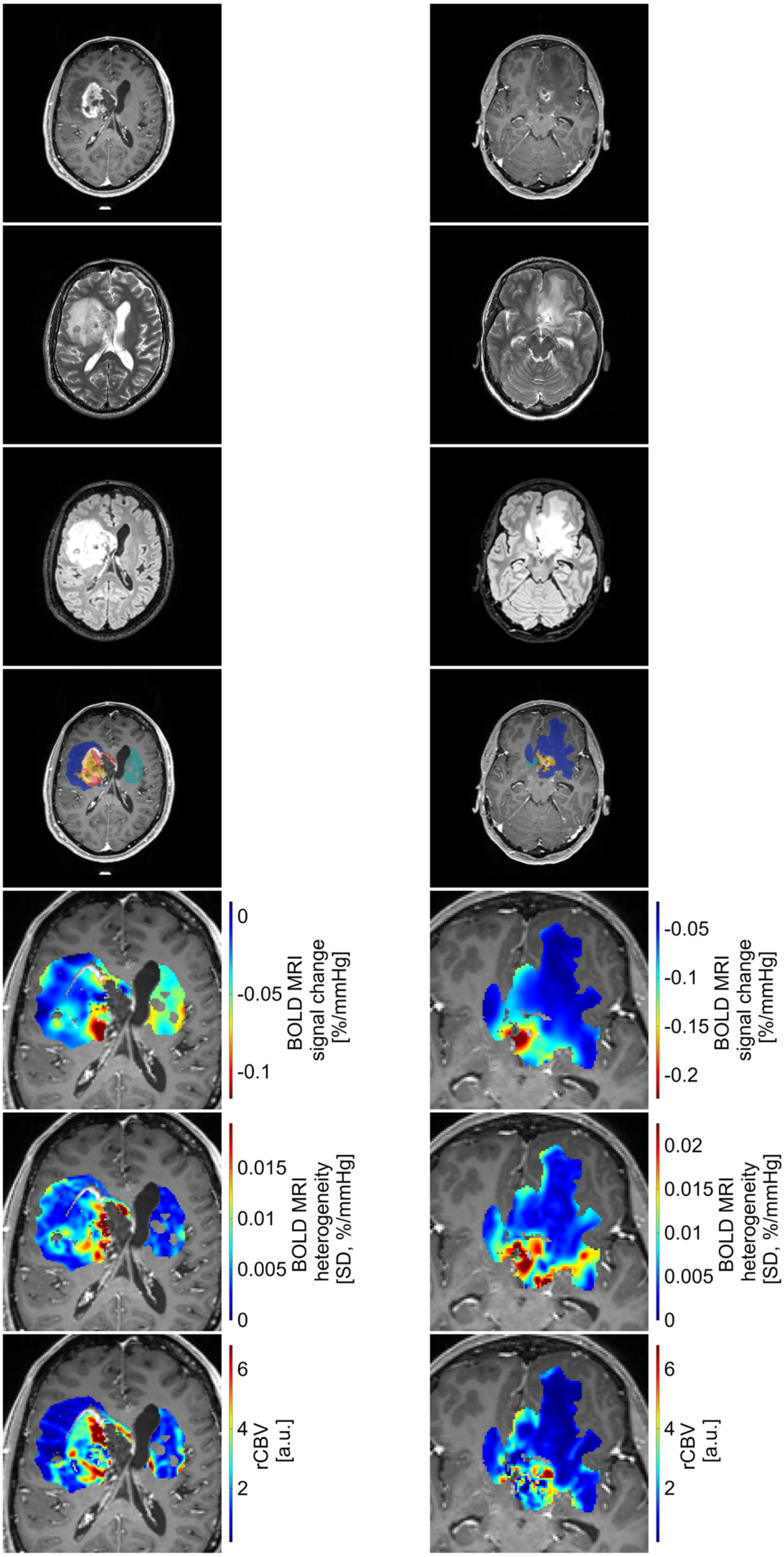
0
-0.05
-0.1
BOLD MRI signal change [%/mmHg]
0.015
0.01
0.005
0
BOLD MRI heterogeneity [SD, %/mmHg]
6
4
2
rCBV [a.u.]
-0.05
-0.1
-0.15
-0.2
BOLD MRI signal change [%/mmHg]
0.02
0.015
0.01
0.005
0
BOLD MRI heterogeneity [SD, %/mmHg]
6
4
2
rCBV [a.u.]
